# Electrical impedance spectroscopy (EIS) in plant roots research: a review

**DOI:** 10.1186/s13007-021-00817-3

**Published:** 2021-11-13

**Authors:** Yang Liu, DongMing Li, Ji Qian, Bao Di, Gang Zhang, ZhenHui Ren

**Affiliations:** 1 College of Mechanical and Electrical Engineering, Hebei Agricultural University, Baoding, 071001 People’s Republic of China; 2 Department of Computer Application Engineering, Hebei Software Institute, Baoding, 071000 China; 3 College of Horticulture, Hebei Agricultural University, Baoding, 071001 China

**Keywords:** Electrical impedance spectroscopy (EIS), Plant root, Root electrical characteristics, Equivalent circuit

## Abstract

Nondestructive testing of plant roots is a hot topic in recent years. The traditional measurement process is time-consuming and laborious, and it is impossible to analyze the state of plant roots without destroying the sample. Recent studies have shown that as an excellent nondestructive measurement method, although electrical impedance spectroscopy (EIS) has made great achievements in many botanical research fields such as plant morphology and stress resistance, there are still limitations. This review summarizes the application of EIS in plant root measurement. The experiment scheme, instrument and electrode, excitation frequency range, root electrical characteristics, equivalent circuit, and combination of EIS and artificial intelligence (AI) are discussed. Furthermore, the review suggests that future research should focus on miniaturization of measurement equipment, standardization of planting environment and intelligentization of root diagnosis, so as to better apply EIS technology to in situ root nondestructive measurement.

## Background

The root is a vital organ of a plant. It is responsible for anchorage, obtaining nutrients in the soil, and improving soil composition [1]. Therefore, it is very important to detect and evaluate the root system of existing plants, which has great development significance [2].

The root system is an important organ for plants to absorb nutrients from the soil. It can synthesize the essential living matter of plants. Plant roots can absorb water and nutrients, affecting crop yield and quality [3–5]. The study of the root system is of great significance to plant nutrition and physiology [1, 5–7]. To improve the utilization rate of water and fertilizer and ensure crop quality and yield in agricultural production, it is essential to accurately obtain root system quality [8, 9]. For decades, roots play a central role in water balance and nutrient cycling in the soil–plant-atmosphere continuum [3].

In recent years, global environmental problems such as climate change, water resources management, and soil pollution have become increasingly prominent, which also promotes research on roots and their functions [10].

### Current status of root detection methods

The research progress of root detection and plant root growth process is relatively slow, which makes it impossible to effectively carry out root research and early prevention of root diseases. Traditional root system research methods, such as the excavation method, soil coring method, and section method, are not only complicated but also time-consuming and laborious. There is a certain degree of human interference and destruction on the original living environment of the root system during the sampling process, which makes the accuracy of the research results questionable [11]. In recent years, a series of root nondestructive testing (NDT) methods such as micro root canal system, Ground penetrating radar (GPR) technology, nuclear magnetic resonance (NMR) imaging method, isotope, and X-ray scanning analysis system has been developed along with a high-density resistivity method [12]. However, all of the methods mentioned above have certain limitations. With the development of root system research, it is particularly important to seek research methods that can accurately detect large and complex roots buried in soil [13]. With a large number of applications in plant physiology and plant pest warning research, bioimpedance technology in botany ushered in a climax.

### Research progress of bioimpedance methods in roots

Bioimpedance technology originated from medical pathology and was gradually applied to many research fields. In the 1980s, scientists began to use impedance spectroscopy measuring technology (ISMT) to study the electrical response characteristics of linear circuit network frequency [14–17]. After the introduction of electrical properties into the research field of biological tissues and organs, the measurement method of bioimpedance spectroscopy has been gradually formed. The electrical impedance method, a new technology developed in recent years, measures the root electrical characteristics (Capacitance, Resistance, and EIS) in the circuit under an external power supply of a certain frequency. Not only is there a good correlation between the electrical characteristics and root biomass and morphological indexes, but this method is also quick to detect and easy to carry out.

#### Single-frequency electrical capacitance method

The theory that plant tissues possess electrical properties such as resistance and capacitance due to cell membranes has been recognized for a long time. In 1965, Walker [18] tested the capacitance theory of plant roots by hydroponic maize. They found that there was a strong linear relationship between root capacitance and the number of root at the nutrient liquid level and its reciprocal.

In 1970s, Chloupek [19, 20] found that there was a good linear relationship between root capacitance value and root quality and morphological indexes of maize, onion and sunflower. Subsequently, the linear relationship between plant root capacitance and root characteristic parameters has been widely confirmed. In 2010, Chloupek et al. [21] analyzed the factors affecting the linear relationship, and concluded that the constant term was affected by the parasitic capacitance generated by soil properties and connecting wires, etc. The relationship between plant root capacitance ($C$) and root size ($x$) can be expressed by the equation: $C = a + bx$, where $b$ is the regression coefficient affected by plant species, measurement frequency, voltage, etc. Although the coefficient of independent variable depended on plant species, it was also affected by the frequency and voltage of the external power supply [19–21]. However, according to Dalton [22], the change of root capacitance due to the change of water should also be considered in the change of root capacitance, and it is suggested that not only the morphological characteristics of roots but also the electrical properties of roots should be considered in the measurement of root capacitance.

In 1995, Dalton [22] first proposed to explain the theory of root capacitance method in the form of equivalent circuit diagram. The model takes into account the electrode soil interface, root medium, root, stem and electrode stem interface, which are represented by a resistor/capacitor parallel circuit R/C and all R/C series circuits respectively. He believed that the two measuring electrodes could be analogous to a parallel circuit formed by a number of capacitors connected in parallel, and only the active root system with absorption function could conduct current. Therefore, the root capacitance characteristics should be the comprehensive expression of the root morphological characteristics and physiological characteristics. In addition, Dalton [22] also found that water content had a great influence on the contact area between roots and soil, which was a key technical problem in the measurement of root capacitance. He suggested that the root capacitance should be measured when soil moisture was close to saturation, and the location of plant electrode in the stem should be close to the root. These important conclusions have become the operating standards widely followed in the later research and application of root capacitance method [20]. In 2005, Rajkai et al. [23] proposed a dual dielectric model, which also takes soil capacitance into account. The root cutting experiment and continuous root leaching experiment conducted by Ozier-Lafontaine et al. [24] have well verified the relationship between root capacitance and root mass in hydroponic media under 1 kHz excitation frequency. Dalton's hypothesis about the dielectric effect that the unimmersed part of the root system does not contribute significantly to the capacitive effect can be clearly demonstrated in the root immersion experiment.

In 2012, in order to verify Dalton model, Dietrich et al. [25] took hydroponic oat as the material and found that there was a good linear relationship between root capacitance value and root biomass by lifting up, cutting off roots in nutrient solution and adjusting the distance between plant electrode and liquid level of nutrient solution, etc. However, when the root system is lifted, there is no linear relationship between the root capacitance and the root biomass in the nutrient solution, but there is a linear relationship between the root capacitance and the sum of the root cross-sectional area on the surface of the nutrient solution. When the roots in the nutrient solution were removed, the root capacitance value did not change significantly, which was completely contrary to the Dalton model. Therefore, Dietrich et al. [25] believed that the root capacitance measured by the root resistance method was actually determined by the composition between the plant electrode and the nutrient liquid level. Dietrich proposed a new model and pointed out that the increase of capacitance value through increasing soil moisture content was mainly soil capacitance [26]. The new model is a series circuit formed by several capacitors connected in parallel. There is a good correlation between the measured root capacitance and the root biomass, but the root capacitance is not the direct measurement result of the root characteristics. Chloupek et al.[21] emphasized that single-frequency root capacitance measurements were only suitable for repeated evaluations of the same plants growing on the same substrate at the same water level and at different ontogenetic stages in the same population, provided that the potting soil water content (SWC) was the same at each measurement time.

I Cseresnyes has made an important contribution to the measurement of single-frequency root electrical impedance. In 2016, I Cseresnyes [27, 28] studied the application of root capacitance measurement technology in the in situ investigation of soybean root growth and drought resistance, and the results of root electrical capacitance (EC) measurement clearly reflected the changes of root growth characteristics and biomass yield of different varieties. In 2018, I Cseresnyes measured root capacitance and SWC of corn and soybean to monitor onto developmental changes in root activity [29]. The results indicated that the root capacitance method could be effectively applied to study the time process of crop root activity in field, and it could also be used to compare the single capacitance data in spatially heterogeneous soil water conditions. I Cseresnyes also measured the impedance phase Angle of the root-soil system of potted wheat, soybean and maize subjected to cadmium pollution, alkali stress, drought or weed competition to monitor plant response to environmental stress [30]. It was proved that the change of impedance Angle measured at a single frequency of 1 kHz in pot experiment was an effective in situ measurement method. In 2019, at a low frequency (1 kHz), I Cseresnyes applied the parameters of capacitance, impedance phase Angle and conductance together for the first time to in situ monitor the root condition of potted wheat [31]. This approach is now being applied to evaluate root responses to different alkalinity levels. In 2020, I Cseresnyes measured the root-soil system capacitors of cucumber, corn, soybean and wheat plants under different soil substrate components, different substrate salinity, and different water content at a single frequency [32]. The results show that the basement impedance and substrate salinity have negligible effects on the capacitance measured in the root-soil system. However, the physical and chemical changes induced by salt may affect the dielectric properties of plant tissues. Capacitance measured in root-soil systems increases exponentially with SWC. I Cseresnyes also used non-invasive root capacitance to monitor seasonal variations and drought response of root activity in perennial *Stipa Borysthenica* and biennial *Crepis Rhoeadifolia* [33]. It was found that there was a strong correlation between root capacitance and root biomass and SWC in a certain period of time, and insufficient natural precipitation and summer drought reduced root activity of both plants. In addition, in order to verify whether the capacitance and impedance measured between the ground electrode and the plant electrode really represented the roots in the soil, I Cseresnyes conducted electrode separation experiment and root excision experiment on potted maize plants cultivated in arenosol. He found that partial electrical separation of grounding electrodes did not significantly change the capacitance and impedance of either the soil or the plant soil systems, suggesting that the root system was dominant in the final measured capacitance and impedance. In the given experimental conditions, it was found that stem base was an important part of root capacitance. This is mainly due to the polarization of epidermal membranes of root neck and the stem base, so the role of stem base cannot be ignored [34]. We summarized the references in the study of in situ root systems using single-frequency capacitance method, as shown in Table 1.

**Table 1:** Summarized references using single-frequency EC method in situ plant root research

Method	References	Plant	Growth substrate	Concern	Frequency range (Hz)	No. of plant
Single-frequency EC method	[18]	Maize	Hydroponics	Root number	1 k	15
	[19]	Maize	Pot Sand	Root wet weight, Root dry weight, Root length, Root surface area	800	24
	[20]	Carrot Sunflower Sunflower	Field Sand Sand	Root wet weight	1 k	113 15 15
	[21]	Carrot	Field	Root wet weight	1 k	92
	[22]	Tomoto	Hydroponics	Root dry weight	1 k	12
	[23]	Sunflower	Pot Sand	Root wet weight	1 k	12
	[24]	Acalypha	Hydroponics	Root wet weight	1 k	5
	[35]	Maize Maize	Pot vermiculite Field	Root wet weight Root wet weight	1 k	32 36
	[25]	Barley	Hydroponics	Root wet weight	1 k	16
	[26]	Barley	Pot chernozem	Root dry weight	1 k	67
	[36]	Bean	Pot Sand	Root dry weight, Root length, Root surface area	1 k	19
	[27, 28]	soybean	Pot perlite	Root dry weight, Shoot dry weight, Root/shoot ratio	1 k	40
	[29]	Maize Soybean	Pot chernozem	Root activity, SWC	1 k	30
	[30]	Wheat Soybean Maize	Pot Pumice or arenosol	Cadmium contamination, alkaline stress, drought, weed competition	1 k	12 20 10
	[31]	wheat	Pot Rhyolite and vermiculite	Salinity-alkalinity stress	1 k	20
	[32]	Cucumber Corn Soybean Wheat	Pot pumice, arenosol, chernozem, pumice	Salinity-alkalinity stress	1 k	30
	[33]	Stipa borysthenica Crepis rhoeadifolia	Sand Calcaric arenosol	Root activity, SWC	1 k	12
	[34]	Maize	Pot arenosol	Root dry weight, stem cross-sectional area	1 k	12

In general, alternating current with a frequency of 1 kHz flows along the root tissue through two circuits: primarily through the extracellular matrix (usually resistive), and rarely through the cell membrane (capacitive) and intracellular space (also resistive) [30]. This is mainly due to the low frequency of the power supply, so most of the current can’t penetrate the cell, so the characteristics of the intracellular resistance and extracellular resistance can’t be studied.

As can be seen from Table 1, root capacitance measurements are usually made with a fixed external AC supply frequency of 1 kHz. In 2017, Peng conducted a linear fitting between root capacitance value and root surface area at multiple excitation frequencies, and found that ${R}^{2}$ was the highest at 1 kHz. Therefore, 1 kHz excitation frequency is recommended as the appropriate frequency for measurement. When the power frequency is greater than or equal to 1 kHz, the influence of soil capacitance on the measurement results can be ignored [37]. The use of this frequency may be attributed to changes in the dielectric constant that occur at the water–air interface [23].

#### Multi-frequency electrical impedance method

With the improvement of electrochemical knowledge and measurement techniques, as well as the wide application of biological resistance method, the method of multiple-frequency measurement has been tried to be applied in the field of root research [38]. While initial studies focused on resistance and capacitance measurements at a single frequency, recent studies have increasingly focused on impedance measurements at multiple frequencies; this approach allows the simultaneous study of conduction and polarization effects, providing information about roots at different temporal and spatial scales [39–41]. Now, multiple frequency measurement methods mainly include EIS, SIP and EIT. One of the most important is the EIS technique, and SIP (spectral induced polarization) is a variant of the EIS used in geophysics, while EIT is used in electrical impedance tomography (EIT).

Nowadays, the EIS method has been tried to be applied in the field of root system research, especially used to evaluate the response of plant organs or tissues to freeze–thaw damage, cold acclimation, osmotic stress, root hypoxia or nutrient deficiency [42–45]. Environmental stress causes changes in the electrical properties of plant tissues by altering many features of the cell wall, cell membrane, and cytoplasm [44, 46]. When the plant is under stress, using the multi-frequency measurement method and the equivalent circuit model, the intracellular resistance, extracellular resistance, intracellular resistivity, extracellular resistivity, relaxation time and relaxation time distribution coefficient can be obtained, so that the stress state of the plant cell, extracellular plant and cell membrane permeability can be quantitatively reflected. However, such rich and detailed plant physiological parameters cannot be obtained by single frequency measurement. These studies focus on the use of laboratory instruments to analyze spectral measurements of reactance and extracellular or intracellular resistance over a wide frequency range (from 5 Hz to 1 MHz).

The research on plant roots can be divided into two types: ex situ measurement technique and in situ measurement technique. The following highlights the use of EIS in situ root measurements, challenges and future developments.

## EIS measurements of root system

### Principal of EIS

**Fig.1:**
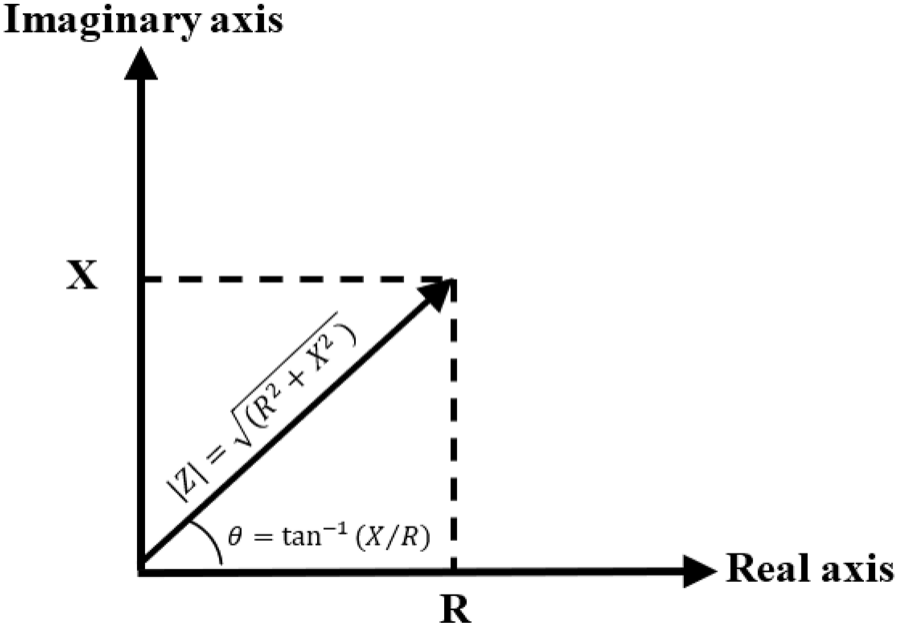
Vector diagram of complex impedance. Z, impedance magnitude; R, real impedance; X, imaginary impedance; $\theta$, phase angle

Bioelectrical impedance ($Z$, Ohms) is the ratio of voltage ($Vsin(\omega t)$) and current ($Isin(\omega t+\theta )$, where the angular frequency $\omega = 2 \pi f$, $t$ is time and $\theta$ is the phase Angle) of biological tissues under a certain frequency (f) AC power supply [47]. This reflects the characteristics of the total resistance ability of the tested biological tissues to the current. Electrical impedance in the form of a complex number vector ($Z=R+jX$), consists of the resistive part ($R=Zcos\uptheta$, Ohms) of the real resistor and the capacitive part ($X=Zsin\uptheta=1/(\omega c)$, Ohms) of the imaginary capacitor, as shown in Fig. 1. The impedance modulus represents the absolute value of the impedance ($|Z|=\sqrt{({R}^{2}+{X}^{2} )}$) and multigroup impedance and capacitive reactance values obtained at a series of current frequencies are plotted on the real axis and the virtual axis of the impedance composite vector plane to form the trajectory of impedance varying with frequency, which is called Electrical Impedance Spectroscopy (EIS) [48–50].

When the alternating electric field is applied to the plant tissue, alternating current (AC) will cause polarization and relaxation phenomena, which will cause the amplitude and phase of the AC signal to change. According to these changes in electrical parameters, the impedance of the plant tissue can be determined. This alternating electric field is usually applied over a specific frequency range to produce a measured spectrum [43]. Due to the passive electrical properties of plants, the tissue impedance of plants is related to cell ion content, membrane structure, and viscosity [51]. With the change of electric field frequency, the electrical characteristics of biological tissues will change accordingly. These findings make it possible for electrical impedance measurement to be applied in many scientific fields of plant research.

In recent years, EIS measurement technology has been continuously developed and improved, which has been widely used in various kinds of stress [44, 52, 53] and has achieved good results in estimating plant activity [54], monitoring nutrient status [55], detecting internal damage of fruit [56, 57], detecting tissue damage [56], and studying plant cold resistance [43, 58–60] along with other aspects.

### Measurement scheme design

Zhang selected potato tubers and carrot roots as the research objects. Before the experiment, the middle segment of potato tubers and the cortex fragments of carrot roots were cut off. After freeze-thawing at − 3 ℃, − 6 ℃, − 9 ℃ and − 12℃, the impedance was measured with computer-controlled Agilent 4284A [61–63]. In experiments designed by several scholars, they all randomly selected 6 to 12 sample seedlings from each treatment group, and a 15 mm root segment was cut from the middle of the main root of the seedling for electrical impedance measurement. They use Ag/AgCl electrode in the test and electrode gel was added to the electrode. The impedance analyzer needed to perform open-circuit calibration and short-circuit calibration before formal measurement and then the resistance and reactance values of the samples were measured using LCR (Inductance, Capacitance, Resistance) instrument [64–67].

As seen from the papers above, the researchers used a destructive sampling of plant roots and cut the roots into several lengths as test materials for the ex situ root EIS measurements. Before measuring the EIS, root washing, drying, freezing, and so on were required. Moreover, to remove the polarization effect, the gel was added on both sides of the root segment during the measurement. The measurement process was not only complicated and required a long time, it was also extremely destructive to the root system, so subsequent continuous monitoring of the root system could not be carried out.

When EIS technology was introduced into the field of plant root research, various characteristics of plant roots can be studied non-destructively when the electrical impedance values are reflected at different frequencies. However, there are not many papers using the EIS method to study the characteristics of the whole root [23, 24, 40]. The main reason is that it is very difficult to measure the in situ root electrical impedance*.* Measurement results will be affected directly by soil composition, nutrient solution ratio, or matrix type, but in situ root measuring has very important significance for root system quality judgment, and in the literature of recent years, in situ root measurement techniques have been reported many times.

During the measurement of in situ roots, all researchers established an electrode–stem–root–soil or nutrient solution–electrode circuit loop, a safe AC power input, and the measurement end of the voltage between two points [24, 38, 39, 68]. Most researchers use a two-electrode measurement system [39]. One electrode (plant electrode) is inserted into the test plant stem, usually 2 mm above the root–soil junction [24, 38, 49, 69, 70], another electrode (soil electrode) is inserted in the soil of plants growing [38]. In the study of Ozier-Lafontaine, a soil electrode was inserted at 10 cm from the stem at a depth of 50 mm [24]. In a study by Cseresnyés, a soil electrode was inserted 10 cm deep into potting soil and 6 cm from the root [71]. In the study by Vozary [53] and Jocsak [44] on flood and cadmium stress in pea seedlings, in situ root impedance measurement was also performed using EIS. The measuring electrode was inserted into the stem 2 cm above the base along the longitudinal axis of the seedling or into the root 2 cm below the base along the longitudinal axis of the seedling, and the distance between the two electrodes was 2 mm. In the study by Repo and Di, the soil electrode was connected to a stainless steel plate electrode, which was serrated and protruding through a hole in the bottom of the pot into the soil substrate [49, 69, 70]. When the root grows in a nutrient solution, another electrode (nutrient solution electrode) is placed in the solution [23, 40] or at the bottom of the solution container [72]. Then the two electrodes are connected to the impedance analyzer. Impedance analyzers can produce single or multiple frequency sin wave excitation voltage signals. The vector voltage and vector current between the two electrodes can be adjusted and then the measured signal vector collected can be decomposed to obtain the projection of current and voltage on each coordinate axis. Then, various electrical parameters and their frequency responses between the two electrodes can be accurately estimated [38]. But the environmental factors such as soil type, SWC, electrode spacing and location, and water content of the root affecting the measurement of EIS are important during root development. When we apply the EIS method to measure the electrical impedance of plant roots, we need to find an appropriate measurement scheme, eliminate the influence of objective environmental factors, and find the internal relationship between plant roots and the measurement results of electrical impedance.

We summarized the references in the study of root systems in situ using EIS methods, as shown in Table 2, which shows the research materials, growth substrate, frequency range, and other related contents reported in the above literature.

**Table 2:** Summarized references using EIS method of in situ plant root research

Method	References	Plant	Growth substrate	Concern	Frequency range (Hz)	No. of frequency	No. of plant
Electrical Impedance Spectroscopy (EIS)	[39]	Willow	Hydroponics	Resistance, capacitance	40 ~ 340 k	488	64
	[24]	Tomato	Potting oxisol	Resistance, capacitance	10 ~ 1 M	51	12
	[73]	Sitka spruce	Field	Resistance, capacitance	40 ~ 340 k	–	18
	[68, 72]	Willow	Hydroponics	Resistance, capacitance	60 ~ 60 k	31	15
	[71]	Maize	Potting Arenosol	Resistance, capacitance, Phase Angle	100-10 k	55	18
	[70, 74]	Scots pine	Potting Perlite	Resistance, capacitance	5 ~ 100 k	44	72
	[69]	Scots pine	Potting Perlite	Resistance, capacitance loss factor δ	80 ~ 1 M	42	630
	[44]	Pea	Hydroponics	Resistance, capacitance, Phase Angle	800 ~ 1 M	100	30
	[53]	Pea	Potting Perlite	Resistance, capacitance, Phase Angle	30 ~ 1 M	100	30–40

As can be seen from Table 2, in the cultivation mode, potted and container cultivation are mainly used, and these two modes are more suitable for laboratory cultivation and research. Crop growth environment mainly adopts nutrient solution and substrate. The composition of nutrient solution can be controlled artificially and easy to be standardized, so that the electrical impedance measurement results have less impact on the root system. However, soil is rarely used as a growing environment, mainly because of its complex composition, which has a great influence on the electrical impedance measurement results and is not easy to be standardized. Therefore, potting is mainly based on the substrate. The composition can be effectively controlled by the substrate with a fixed ratio, and the interference data can be effectively eliminated by data subtraction [43, 53, 75, 76].

### Using of experimental electrodes

The electrode acts as a transducer between ion transport in the biological tissue and the flow of electrons on the wire. The flow of ions in the electrolyte results in the flow of electrons in the electrode (or vice versa) [24]. Measurements of materials in the frequency range of 10 Hz to 1 MHz are affected by electrode polarization and cable inductance. Open and short circuit correction can be effective for their effects [43].

**Fig. 2:**
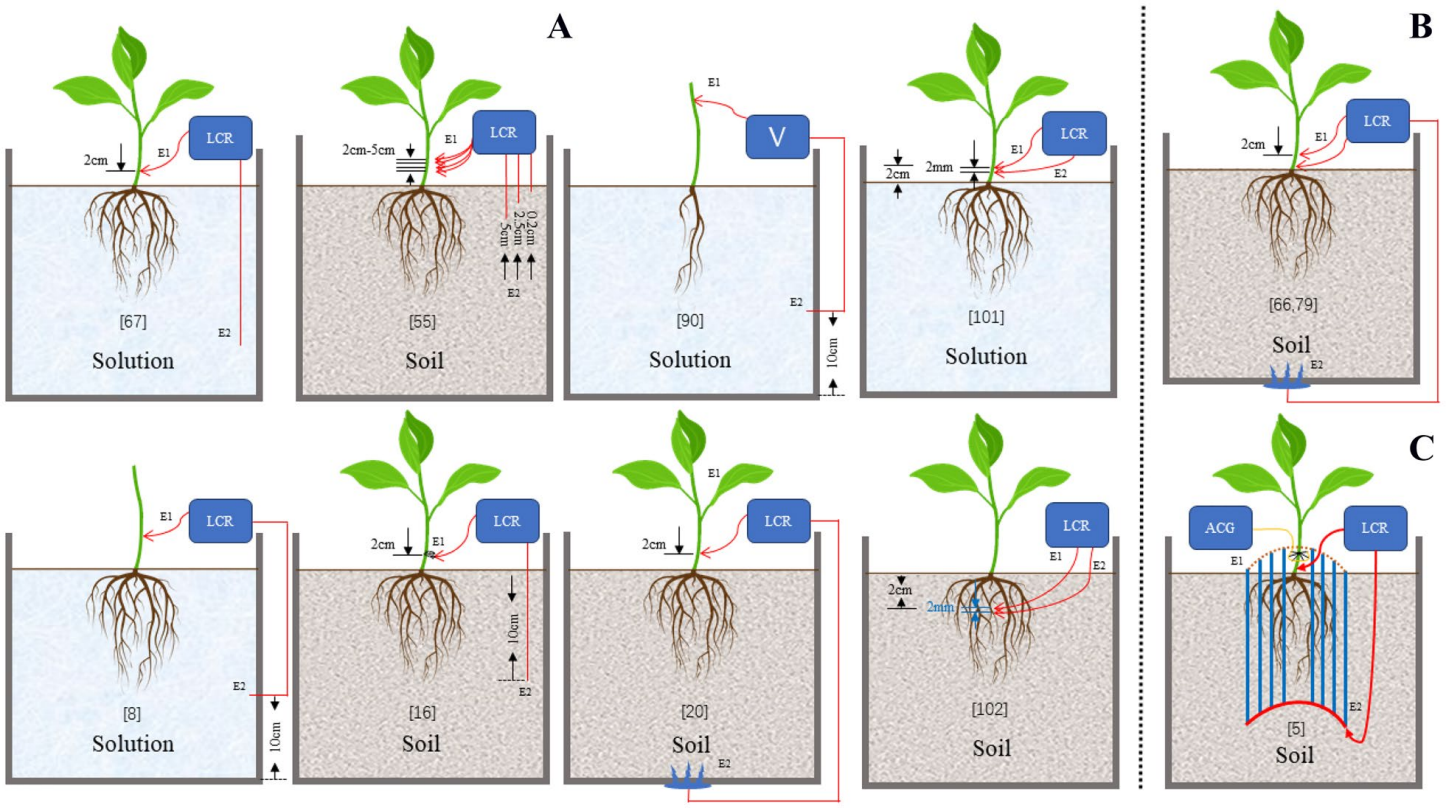
Different electrode positions and measurement schemes. **A** Two-electrode measurement scheme. **B** Three-electrode measurement scheme. **C** Multi-electrode measurement scheme. ACG: Alternating Current Generator. V: Voltmeter. LCR: Inductance, Capacitance, Resistance

**Fig. 3:**
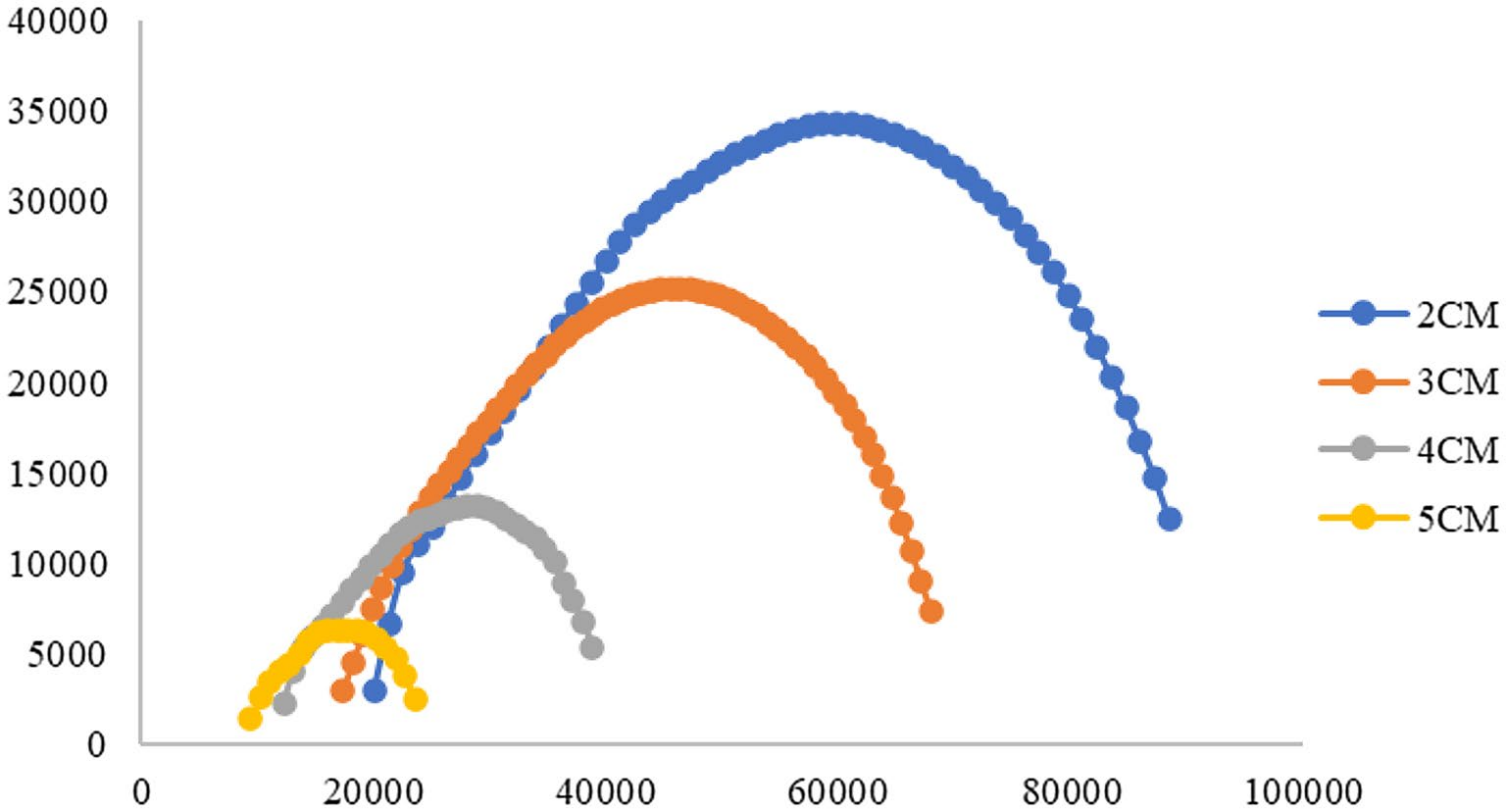
The change in resistance and reactance as the different placement of the plant electrode from the base of the stem to 5 cm height. (Fig. 3 from reprinted Ozier-Lafontaine and Bajazet [24], reprinted with permission of the author)

The literature on in situ measurement of root electrical impedance shows that the electrode types are mainly divided into needle electrode and clamp electrode. Needle electrodes and clip-on electrodes were used in a pot experiment on sunflower plants. It was found that when the medium was water, clamp electrode could be used, while in other media, needle electrode had better correlation with root growth [23]. In 2005, Rajkai performed EIS measurements using an HP4284A instrument at an external excitation voltage of 1 V in the frequency range of 30 Hz ~ 1 MHz. Hypodermic needle and spring tension clamp were used as two types of plant electrodes in the measurement process. The physical size and geometry of the clamp's tip of the spring tension clamp is not important because the conductive gel applied determines the effective surface area of the plant electrode. In the same species, growth stage, temperature and humidity of the external environment, the measured impedance of the clamp electrode is 150 Ω higher than that of the needle electrode, and the measured capacitance is 2 nF lower than that of the needle electrode. Moreover, the polarization ability of the clamp electrode is significantly weaker than that of the needle electrode. With water as the root medium, clamp type plant electrode can be used instead of needle electrode [23]. In terms of electrode design, most plant EIS measurements were made using a Dual-terminal (2 T) configuration with Stainless steel electrode or Ag–AgCl electrode to reduce polarization effects. Generally, one end of the electrode is inserted 2–5 cm above the junction of the rhizome [24, 77] and the other end is designed as a stainless steel plate electrode and inserted into the bottom of the pot [49, 69, 70, 78]. The other end could also be made into a rod electrode and inserted into soil or hydroponic solution to form a conductive circuit and achieve the measurement of electrical impedance at different frequencies. Figure 2 shows different electrode positions and measurement schemes. It can be seen that the two-electrode configuration is the most commonly used, with a simple circuit and strong operability. Figure 3 shows the change in resistance and reactance as the different placement of the plant electrode from the base of the stem to 5 cm height.

With regard to the height of plant electrode insertion, Ozier-Lafontaine & Bajazet confirmed the results of Dalton [22] that when the electrodes were inserted far away from the base of the stem, the size of the EIS increased significantly, and the root impedance spectrum and root capacitance decreased. This suggests that great care must be taken when manipulating the plant electrode, and that the insertion position of the electrode must be consistent to avoid greatly affecting the impedance measurement results. Furthermore, the electrode should be inserted as close to the first lateral root as possible [22]. The characteristics of these electrodes were also mentioned in the papers of Chloupeck [20] and Van Beem et al. [35]. In addition, Ozier-Lafontaine & Bajazet also found that when measuring EIS in the field, the depth of the soil electrode placement, distance from the plant, and position had residual effects.

On the other hand, the depth of needle insertion into the stem should be different from plant to plant for EIS measurements. This is mainly due to the difference in resistance between phloem and xylem of plant stems [79]. For woody plants, current flows mainly along the xylem and then through the deeper woody roots with minor leakage [80]. The electrodes are usually inserted in the middle of the stem base. In herbaceous plants, the xylem is less developed and contains few lignified cells, so inserting an electrode needle in the middle of the stem, or even directly, can make the conducting circuit more stable.

To sum up, we can see the basic scheme, cultivation mode, electrode type, and electrode placement of in situ root measurement through relevant literature. In the measurement process, a large special impedance analyzer is used. Based on different excitation source frequencies, the samples are measured only once to obtain the electrical impedance values at different frequencies. Then, the electrical impedance values are analyzed and processed to find the correlation with the research target. The above measurement scheme is simple and feasible and has high requirements for equipment. The measurement can only be completed in the laboratory. With the development of EIS technology, the demand for in situ impedance measurement of samples and uninterrupted continuous impedance monitoring begin to appear. If possible, in situ impedance monitoring can be performed anywhere, either in the laboratory, in the choice of hydroponic solution culture, substrate culture, or soil culture with specific components, or the field. We also can undertake timing electrical impedance monitoring, in this way, the study of plant samples in a certain period (a day or a month) continuous electrical impedance change of values will be obtained, and applied to the plant physiology research and the environment, studies on the adaptability of the EIS technique application solutions will be the emergence of new research results, lays the foundation for the further research of plant EIS technology. The further application of EIS technology must require a breakthrough in miniaturization, convenience, portability, and low cost of EIS measuring equipment on the premise of maintaining accurate measurement data.

### LCR instrumentation

**Fig.4:**
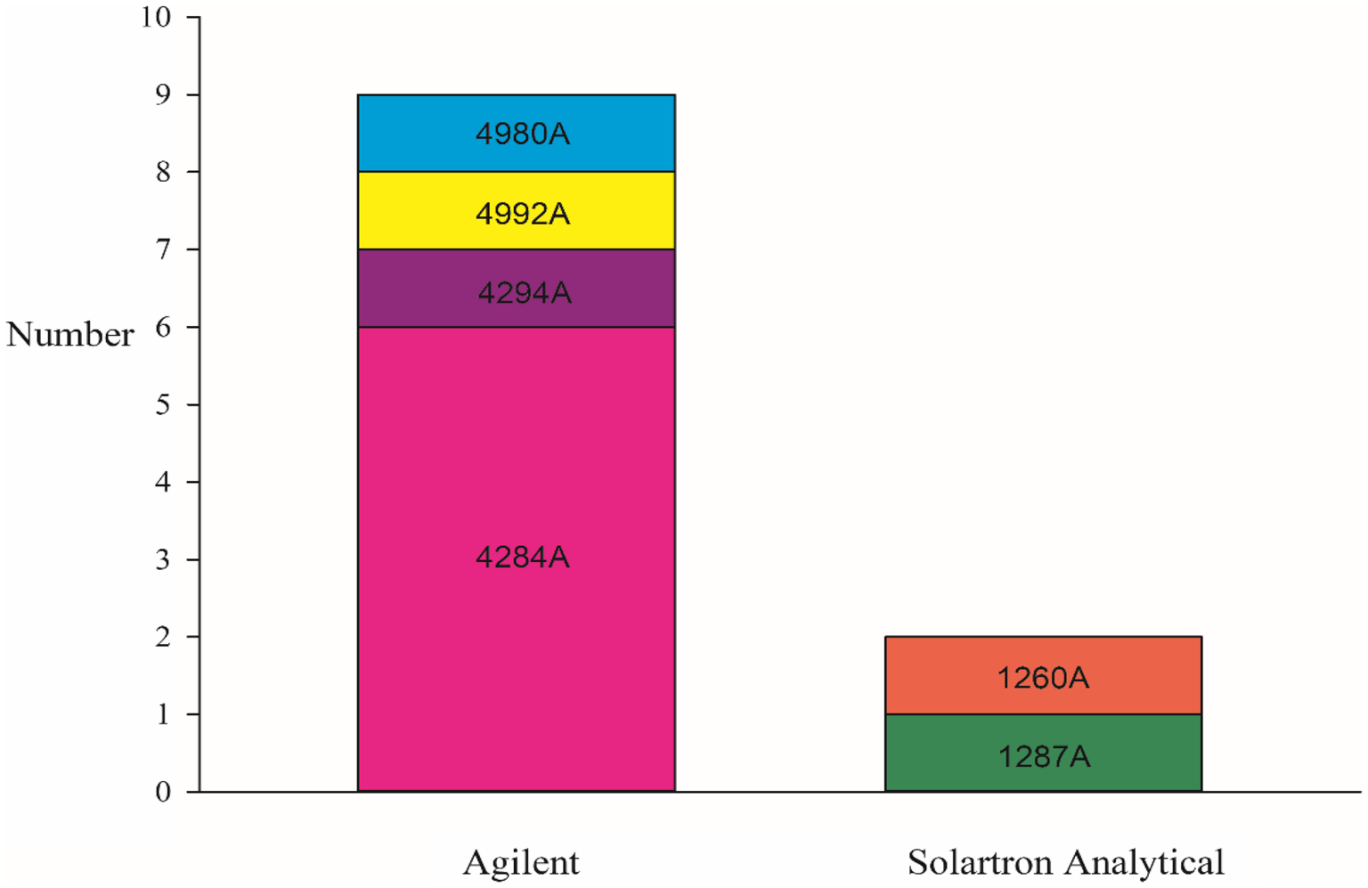
The number of electrical impedance instrument applications

An impedance measuring instrument is the key instrument of plant EIS measurement scheme, its parameters and performance determine the results of impedance measurement and the level of reliability. Modern impedance analyzers can measure the impedance and phase angle of samples at different frequencies [81]. In the published literature, we found several types of impedance testers. After analysis and sorting, we found that the impedance testers used in scientific research mainly came from two manufacturers. One is the Solartron 1260A, 1287A by Solartron Analytical and the other is the impedance analyzer of Agilent, such as 4284A,4294A,4992A LF, and E4980A. As seen in Fig. 4, the instrument of Agilent, especially the impedance instrument 4284A, is the most favored by researchers, with the use ratio reaching 81.8%. However, the measurement process of these instruments is relatively complex, the detection speed is slow, the instrument mobility is poor, and it is difficult to achieve field measurement.

In recent years, there are already high-precision, low-cost impedance measurement chips on the market, such as the AD593x series, AD594x series [82, 83], AD983x series [84, 85], ADuCM350 series [86]. The electrical impedance measurement system can use a high-performance processor as the control core, and a high-precision impedance conversion chip mentioned above as the measurement core. The parameters of the impedance measurement chip can be set through the control core, and the multi-channel multi-frequency automatic measurement can be completed with the optimization of the internal algorithm. Satisfactory results can be obtained by comparing and correcting the measurement results with the instruments of the above two manufacturers. Therefore, we can develop a portable, stable, and accurate impedance measurement system according to the actual research needs, and provide hardware support for the extensive application of EIS technology in botany.

### Excitation source frequency range

According to a large body of literature, EIS has been widely used in plant research [87]. In particular, it has become an effective method to measure root morphological parameters, such as root surface area, dry weight, and wet weight, etc., and it has also been applied in the measurement of root biomass and activity [22, 68, 71, 88]. In many research processes, the frequency of excitation source is selected from both low and high frequency regions, and the measurement frequency used in different plant parts is not the same.

In published research, EIS in the lower frequency range (10 Hz ~ 1 MHz) has been widely used in medicine and plant science [89]. In botany, electrical impedance is usually measured at two frequency scopes: low frequency (50 Hz ~ 1 kHz) and high frequency (100 kHz ~ 1 MHz) [90, 91]. In plant cell studies, low-frequency currents only move along cell walls, while high-frequency currents can penetrate membranes and intracellular fluids [81].

During the measurement of the ex situ electrical impedance of roots, EIS monitored the root state of plants by scanning within a given frequency range, The electrical impedance of potato tuber and carrot root cortex in the range of 100 Hz ~ 800 kHz was measured before and after freeze–thaw cycles [62, 63], measuring pM-ATpase activity and studying the relationship between electrical impedance (measuring in vitro roots at 80 Hz ~ 1 MHz) [65], Cd stress (measuring in vitro roots at 80 Hz ~ 1 MHz) [67], waterlogging resistance (measuring in vitro roots at 80 Hz ~ 1 MHz) [66], cold tolerance (measured in vitro stems and roots at 80 Hz ~ 1 MHz) [43] and salt tolerance (measured in leaves at 5 Hz ~ 130 kHz) were measured at early stress diagnosis.

**Fig.5:**
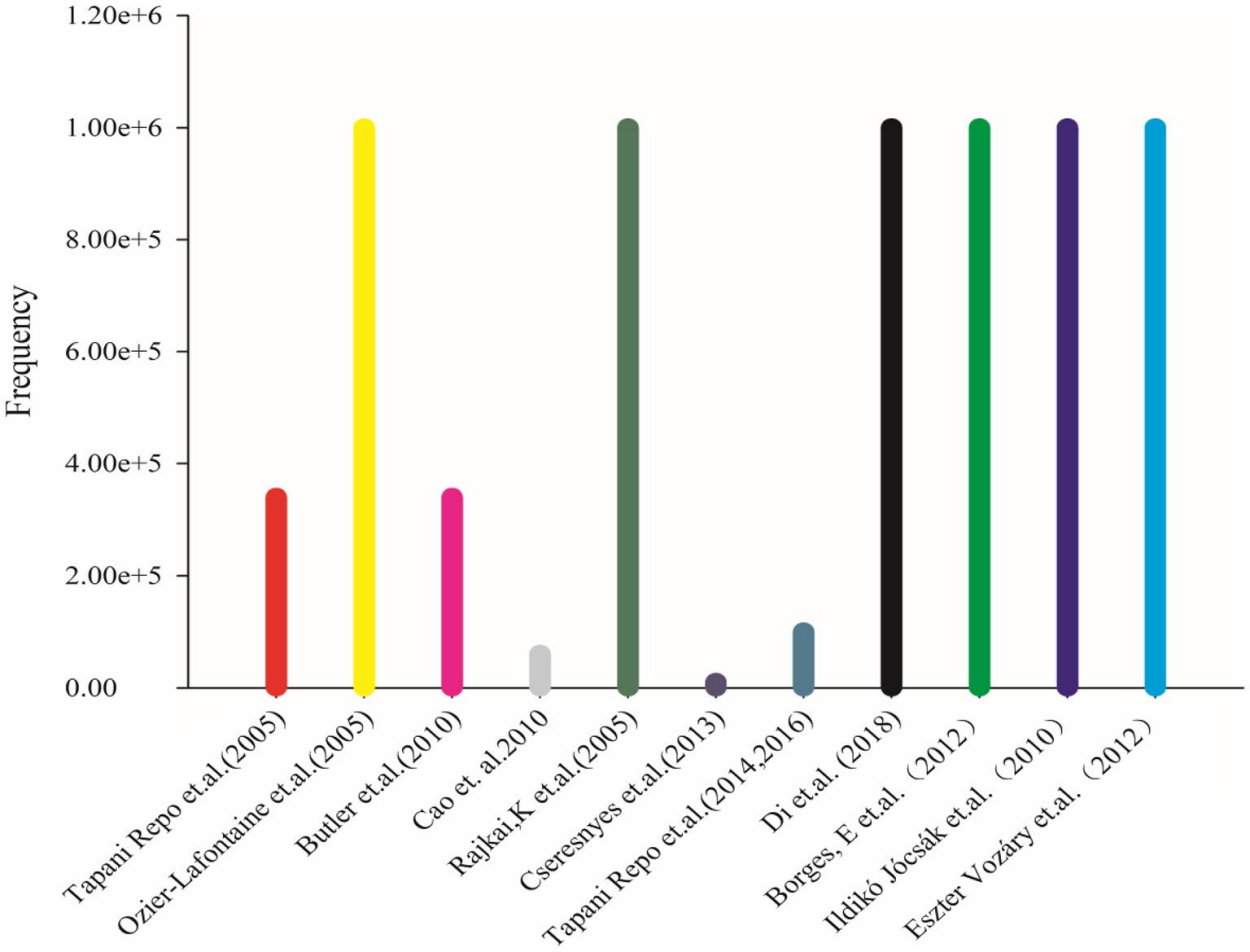
The range of excitation frequencies used in the literature report

There is a great difference in the frequency of EIS measured with in situ roots. According to the different application scenarios of electrical impedance measurement, the researchers will conduct multiple experiments to make the different frequency of excitation power supply design for different application scenarios, and has reached the optimal detection result; we statistics the frequency distribution of root system in situ measurement, the frequency range of differences application scenarios can be seen from the Fig. 5.

The data in Fig. 5 shows that the frequency selection of impedance spectrum varies greatly, and different excitation frequency ranges are selected for different applications. The excitation frequency used in these studies covers the range of 0 ~ 1 kHz, that is, the excitation in the low-frequency band, while the frequency of 1 kHz is very special, which is recognized by scholars as to the working frequency of the excitation source to break down the cell wall and is of great significance in the impedance spectrum research. The highest reported frequency of measurement is 1 MHz, at which the current is already flowing between cell membranes, and any higher frequency is of little significance.

## Root electrical characteristics under EIS excitation

EIS can be used to identify and track plant root cell responses, both in vitro and in vivo [92, 93], and is a non-invasive technique that can provide wide frequency range measurements [94]. The characterization of conductive metal coating films by EIS shows that the low frequency measurement only reflects the characteristics of the film, while the high frequency measurement reflects the characteristics of the film and the solution [95]. The study of root electrical properties and root current pathways is the core content of applying bioimpedance method. The electrical impedance value of biological tissue includes a resistance value and a capacitance value depending on the frequency of the excitation source [96, 97]. The real part of the impedance is related to the resistance path of the current through the tissue, and the imaginary part is related to the capacitance path of the current through the tissue, such as the cell membrane. The real part is dominant at low frequencies and the imaginary part at high frequencies [94].

### Transmission of currents in plants

In the biological sample being tested, the proportion of current passing through the apoplastic and symplastic spaces in the tissue is determined by the magnitude of the AC frequency [43]. Biological tissue cell membranes are composed of dielectric materials with poor conductivity [98]. The lipid bilayers of the plasma membrane can be thought of as capacitors that store energy in an electric field and block the flow of low-frequency alternating current. When different frequency currents pass through biological tissues, the structure composed of extracellular fluid, cell membrane, and intracellular fluid will show different electrical characteristics. As the frequency gradually begins to rise, the cell membrane slowly becomes conductive, which causes a noticeable change in impedance, which can be thought of as a combination of intracellular and extracellular resistances in parallel [24].

**Fig.6:**
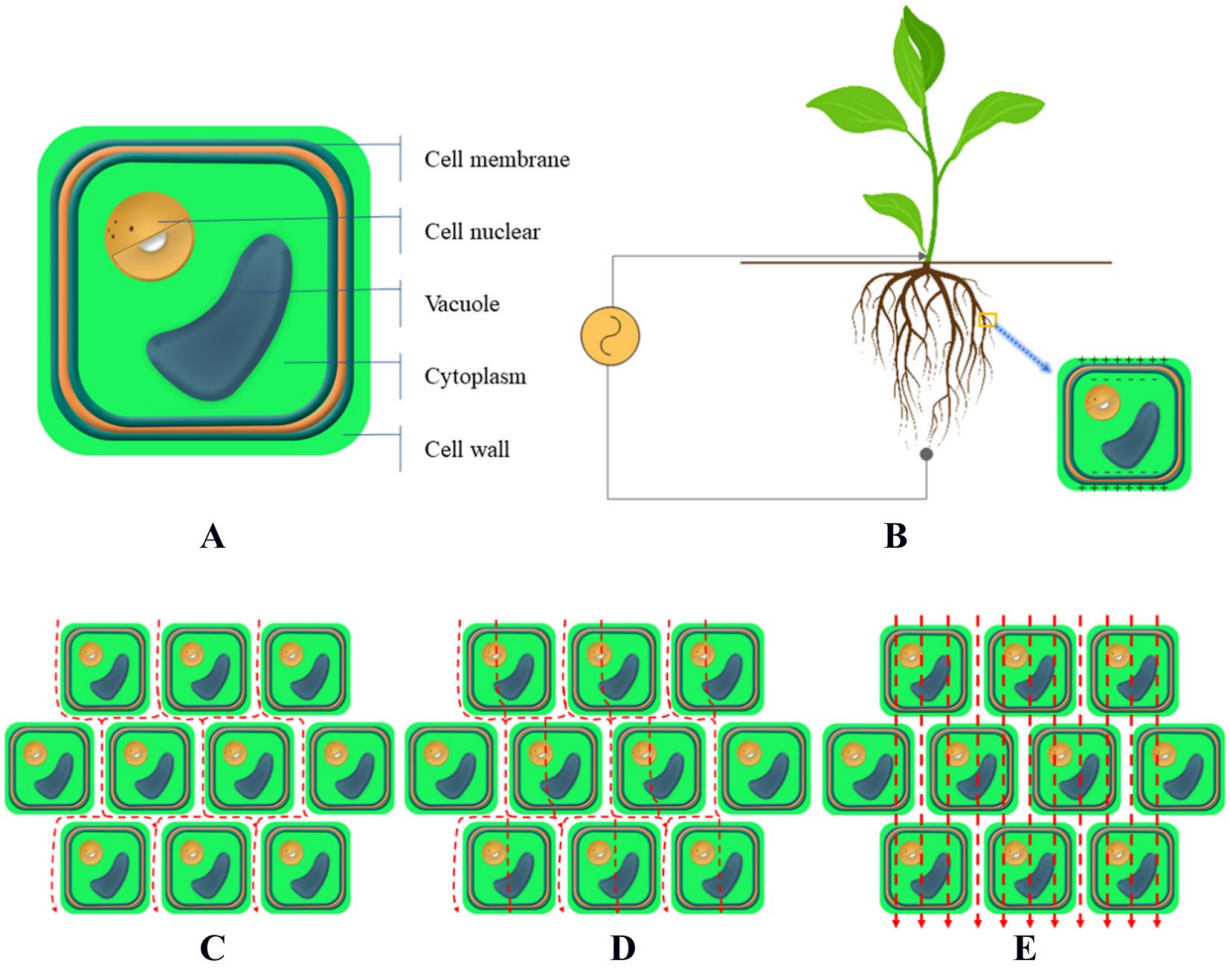
Schematic diagram of **A** plant root cell structure, **B** electrical properties of plant root cells under EIS measurement scheme, **C** low frequency current path, **D** medium frequency current path, **E** high frequency current path

Current conduction in the root depends on the resistance of the extracellular and plasmic bodies, and all membranes and walls play an important role in charge storage (polarization). Since charged particles such as Na^+^, Ca^2+^, K^+^, Cl ions and amino acids cannot diffuse through the cell membrane, polarization occurs on the surface of the cell membrane [41]. When alternating current is applied, the intensity of polarization depends on frequency, intensity and injection time, cell membrane surface charge density, transmembrane potential difference, ion concentration, water content, tissue composition, and tissue health or structural heterogeneity [78, 99, 100]. Polarization may occur both on the external surface of the root (the interface between the root and soil) and in the internal root system [41]. Schematic diagram of root cell structure and electrical properties can be seen in Fig. 6A, B.

Relevant studies have shown that the process of ions passing through cell membranes is mainly controlled by ion pumps and ion channels [41]. At low frequencies, the ion channels of the cell membrane are closed because the low frequency electric field produces small changes in the membrane potential difference, and these changes are too small to change the characteristics of the ion channel [41, 101]. In this case, the impedance of the cell membrane is so high that it does not allow current to pass through. So, the current can only pass through the extraplasmic body, as shown in Fig. 6C. At this low frequency, the total impedance is mainly determined by the extracellular fluid resistance [41, 78, 102].

As the frequency rises, the electric field applied at the outer surface of the membrane changes the potential difference, which opens a partial gate of ion channels and flux across the membrane [101, 103, 104]. Under high frequency excitation, the current thus flows through part of the cell and across different interfaces (Fig. 6D). As the frequency continues to increase, the potential difference eventually opens up all the ion channels, allowing the current to pass through the membranes of all the cells (Fig. 6E), so the total impedance generated will be the combination of the apoplast, extracellular fluid, membrane and cytoplasm [41, 78, 102]. Of course, the frequency at which all the ion channels are opened varies by plant type and plant age. [105]

Urban et al. believed that since most charge carriers passed through the plant tissues near the root neck, the impedance response was mainly determined by the proximal part of the root-soil interface, while the distal fine roots were only slightly affected [106]. In contrast, Ellis et al. showed that most, instead of the entire root length, is electrically connected, so thicker proximal roots have less effect on capacitance. These authors found that stem and soil impedance are primarily resistive, and therefore the root is the primary capacitive unit in the plant root-soil system [36].

Dalton [22] proposed a model to explain the plant root capacitance results in which the current is evenly distributed in the root system. Subsequently, a large number of studies have applied Dalton's model to record the predictive correlation between root capacitance and mass [25]. And recent studies on wheat, soybean and maize roots continue to support Dalton's model. [29, 107, 108].

### Influence of growth media on conductive pathways

Although more and more studies support the capacitance method, some scholars also put forward some different views. They argued that previous roots bioelectrical impedance analysis methods focused on the impedance of plant tissues and ignored the influence of growth media.

The electrical impedance analysis method mainly measures the electrical impedance response of the root system at a single frequency or a frequency range. The measured electrical impedance responses were used to estimate root characteristics such as root absorption area and root mass [24, 25]. The estimation of these root characteristics is based on a key assumption in the electrical properties of roots: that current flows through and is distributed throughout the root system before reaching the soil, and that no current leaks into the soil near the root.

The distribution of leakage current is mainly determined by radial conductivity and longitudinal conductivity and the difference between root and soil resistivity. When longitudinal conductivities are significantly higher than radial conductivities, the current mainly flow to the distal active root through xylem, and the active root is mainly root hair [109]. This is consistent with the root water uptake process, in which the root hairs play a dominant role, while the more insulated and softer roots mainly act as water and current channels. In contrast, when longitudinal conductivities are similar to radial conductivities, currents do not tend to pass through the entire root system, but instead begin to leak from the proximal end of the root into the surrounding medium. In addition, it is well known that soil conductivity is closely related to SWC. As mentioned earlier, SWC can affect root physiology [80].

Plant growing conditions have been shown to affect both water uptake and solute absorption [80]. Moreover, root current path is also affected by growth conditions. [106]. In 2011, Urban found that the tree-root-soil continuum was a series circuit in which xylem and soil resistance was higher than the action of contact resistance [106]. Rao's study [79] found that the most important factor affecting electrical characteristics of roots was the volume ratio between soil and roots, with a correlation coefficient of 0.89, and the lowest correlation coefficient with root surface area and root radius. Redjala [110] observed that the cadmium uptake of hydroponic maize roots was higher than that of aeroponics maize roots. Tavakkoli [111] proved that the salt tolerance of hydroponic barley was different from that of soil barley Enstone and Peterson [112], when studying the effects of hypoxia on maize, reported differences in oxygen flow between hydroponic and vermiculite-grown plants. The observation of Enstone and Peterson [112] could explain the negligible contribution of distal roots to signal under hydroponic conditions [25, 72]. Peruzzo's work showed that, in both soil and hydroponic conditions, percolation of electric current occurred near the root system [80].

Furthermore, Dietrich [25] studied the effect of pruning submerged roots on the electrical impendence response and found that the change in root capacitance was negligible, which was also inconsistent with Dalton's hypothesis. Cao [72] reached a similar conclusion for the measured root resistance. Urban [106] argued that there were numerous determinants of soil and xylem resistivity that interfere with natural systems, and that these factors varied greatly in both space and time. It was found that most of the charge carriers left the root system at the proximal end of the root system, and the electrical impedance measurement method could not provide reliable data on the absorption area of the distal fine root. Peruzzo [80] also experimentally found that current leaves the root channel at its proximal end and radially infiltrates the surrounding growth medium. These results confirm studies that have questioned the assumptions of bioelectrical impedance analysis [25, 106]. However, similar methods using electrical properties have shown that empirical calibration between root electrical properties and root structural properties can determine root biomass nondestructively [19, 22, 24, 35, 107]. However, this empirical calibration requires highly standardized rooting media to minimize differences in root conductivity between soil and individual plants [106].

According to relevant literature reports, there are some different expressions about the effect of growth media on the root conductive pathways. In the root EIS experiment, first of all, the culture matrix for plant growth is formulated in a fixed proportion, which can meet the requirements of standardization. Secondly, plant growth environment does have an impact on the measured values of electrical impedance, and the classical root EIS in situ measurement method does measure roots and growth media together, and the root impedance spectrum obtained is an arc with the real part of impedance as the X-axis and the imaginary part as the Y-axis. We do not need to get the specific impedance value, but through multiple measurements at different times, according to the arc bending changes to judge the degree of environmental stress plants. Or the electrical impedance spectra were measured in the hydroponic solution and correlated with root morphological indexes such as root length or root surface area. Or we can only rely on the electrical impedance data of the root-soil system to classify the roots by algorithm.

The measurement result of electrical impedance will be affected by the difference of cultivation standards. So, our future goal is to measure EIS data on the basis of international standardization (homogeneous planting medium, constant soil moisture, salinity and temperature, consistent electrode placement), and then perform subtraction operations to eliminate the influence of solution and electrode/solution interfaces on the electrical impedance measurements. We pay more attention to the changes in the measured values of the electrical impedance spectra of the root-soil system at different times. The degree of biological stress and abiotic stress are found by AI algorithm.

### Equivalent circuit of the root system

When the plant tissue is placed in a certain AC electric field, a corresponding electric potential difference will be generated when the current passes, indicating that the tissue cells can resist the current [42]. Plant cells contain different cellular structures, each of which has different electrical properties. Vacuoles and cytoplasm resemble resistors, while the cell membrane has the characteristic of capacitance. Therefore, according to the effect of different cellular structures in plant tissue cells on the AC electric field, their response to the current can be described by an approximate circuit, which is called equivalent circuit.

EIS can be used to simulate the internal physiological condition of the measured tissues by establishing an equivalent circuit model, which determines the basic physiological information of biological tissues and organs. When AC is applied to a plant tissue, the proportion of current passing through the extracellular and intracellular spaces varies with the frequency of the alternating current and the nature of the tissue [89]. Studies have shown that the impedance of plant tissues is mainly determined by three factors in the frequency range of 10 Hz ~ 1 MHz: intracellular (coplastic) impedance, Intercellular (extracellular or extracellular) resistance, Cell membrane impedance. However, these impedance data cannot be directly measured and must be fitted in an equivalent circuit to determine the specific value. The cell membrane is like a capacitor whose capacitance value depends on the excitation source frequency [113]. When plant tissue is represented by an equivalent circuit, tissue characteristics can be quantified by equivalent circuit analysis. This analysis of electrical impedance data measured at different AC frequencies is called impedance spectroscopy [114].

#### Equivalent circuit model

Electrical impedance analysis has been widely used in the evaluation of cold resistance and low-temperature damage of plant tissues [43, 58, 59, 62, 63, 115]. The single-frequency approach provides a simplified view of the equivalent model of the system as a whole because it does not consider multiple resistors and capacitors in the circuit [68]. According to the literature on multi-frequency EIS measurements, two equivalent circuits have been used in this application field, one is called lumped model, the other is called distributed model. The lumped model is composed of a finite number of ideal resistors and capacitors (no equivalent inductance is found in plant tissues), each lumped element (resistor or capacitor) corresponding to a biological structure (such as plasma membrane or extracellular space). This kind of circuit was often used in the past [58, 62, 63, 98]. However, the distributed model cannot be represented by a finite number of ideal resistors and capacitors. In a distributed model, the components of each model do not necessarily correspond to a biological structure but may be multiple [58]. Therefore, it is difficult to interpret impedance data from a physiological perspective based on distributed models.

The distributed model is in the form of compact mathematical expressions, which can provide a good fit for experimental data [58]. When the model fits the experimental data better, we can conclude that the parameters of the distributed model are more sensitive to the changes of plant physiological indexes than those of the lumped model. Based on the distributed circuit element (DCE) model, the researchers also found that the resistance in the distributed circuit model decreased with the increase of root mass [43, 116].

There are also obvious differences among plant root tissues. Cells in different tissues are of different sizes and different morphological structures. There are generally four distributed models, all of which are composed of DCE: single-DCE model (including a resistor in series circuit with one symmetric DCE), double-DCE model (including a resistor in series circuit with two symmetric DCE), HN model (including a resistor in series circuit with one asymmetric DCE) and model A [43, 58, 62, 98, 115, 117]. Finally, which model to use depends on the number of arcs in the electrical impedance scatter diagram and the skewness of the spectroscopy. While the number and skewness of map arcs are related to plant tissues and organs, the EIS of the root system generally has one or two arcs, and the thicker root usually has two arcs. Although a biological structure does not correspond directly to each component of the DCE model, they have the advantage that the model can usually take an appropriate mathematical expression, this mathematical expression is well suited to the measured data [58, 75]. The EIS of plant tissues is often described in the following models.

1. **Hayden Model**
  First, we should introduce the Hayden model [113], which includes cell wall resistance (${R}_{1}$), cell membrane resistance (${R}_{2}$), cytoplasm resistance (${R}_{3}$), and cell membrane capacitance.
  The equivalent electrical impedance of the model is calculated as follows: X is the resistance, X = 1/C $\omega$, C is cell membrane capacitance, $\omega$ is frequency and $j=\sqrt{-1}$ if ${R}_{2}\gg {R}_{3} \, and \, {R}_{1}$, then
  1 $$Z=\frac{{{R}_{1}}^{2}{R}_{3}+{X}^{2}{R}_{1-j}X{R}_{1}({R}_{1}-{R}_{3})}{{{R}_{1}}^{2}+{X}^{2}}$$
  If the frequency is lower and $\omega \to 0$, then $Z = {R }_{1},$ and if the frequency is higher, so $\omega \to \infty$, then $Z = {R}_{3}$ [105].
  The improved Hayden model is a more perfect version that takes full account of the fact that cell membrane impedance is frequency dependent [118]:
  The formula of impedance is as follows:
  2 $$Z=\frac{1}{\frac{1}{{R}_{a}}+\frac{1}{{R}_{s}}+{Z}_{m}}$$
  From the above formula, it can be seen that the electrical impedance consists of the extracellular (apoplastic) gap ($Ra$), the intracellular (symplastic) gap ($Rs$), and the membrane impedance ($Zm$) with constant $\varphi$ angle. Where the value of $Zm$ is:
  3 $${Z}_{m}=coscos \varphi +jsinsin \varphi /{C}_{m}\omega $$
  where $j=\sqrt{-1}$, $\omega =2\pi f$, $f$ is the frequency of the excitation source voltage, and ${C}_{m}$ is cell membrane capacity.
  In the study of Jócsák and Vozary, the impedance spectrum of the modified Hayden model was approximated by complex least square method. The modified Hayden model is a relatively simple explanatory model for plant tissues. It includes the extracellular clearance resistance, intracellular clearance resistance and the compound capacitance of cell membrane. The model describes two parallel ways in which electricity can flow in living tissue: one flowing between cells and the other passing through the membrane and inside the cell [44, 53].
  In the distributed circuit model, resistors, capacitors, and inductors can be continuously distributed throughout the circuit. The distributed element model is more defined and complex than the lumped model [119].
2. **Single-DCE Model**
  The Single-DCE model is mainly used to represent the electrical impedance spectrum characteristics of herbaceous root or seedling root. Luo-Sha, Z found that the EIS measured in *Syringa Oblata Lindl*’s roots after cutting was a single arc, which could be used for equivalent circuit construction using the Single-DCE model [65]. Meng, Y. found in the study of Roots in *Betula Platyphylla Suk*. After treatment, root segments were selected for each treatment and the EIS was also a single arc [66].
3. **Double-DCE Model**
  In some cases, impedance measurements in biological tissues often produce impedance spectra consisting of two arcs strongly inhibited by the centers, in these cases, a dual-distributed model can be used to solve the problem [119, 120]. The resistance of the model ($R$,$R1$, and$R2$) can be calculated by the intercept between each circle and the X-axis; the relaxation time ( $\tau 1$ and $\tau 2$) describe the position of the dispersion range at a frequency and are obtained from the vertices of the arc and the vertices of the plane. The distribution of relaxation time is described by the coefficients $\psi 1$ and $\psi 2$ [119]. The double-distributed model considers the vacuole resistance in the cytoplasm and the vacuolar membrane capacitance [62, 63]: where ${R}_{1}$ is the resistance of the cell wall and the extracellular space, ${R}_{2}$ is the resistance of the cytoplasm and the symplasm, ${C}_{3}$ is the cell membrane capacitance, ${R}_{4}$ is the vacuole resistance and ${C}_{5}$ is the vacuolar membrane capacitance.
  Repo found through experiments that the circuit between the two electrodes consisted of a stem, the interface between roots and shoots in the culture medium, the culture medium, and the contact layer on the electrode surface. The impedance spectrum of the system is modeled by a circuit consisting of a resistor, two ZARC-Cole elements, and a constant phase element in series [39]. The complex impedance (Z) of this circuit is calculated by the following formula:
  4 $$Z=R+\frac{{R}_{1}}{1+{(j{\tau }_{1}\omega )}^{{\psi }_{1}}}+\frac{{R}_{2}}{1+{(j{\tau }_{2}\omega )}^{{\psi }_{2}}}+\frac{1}{{{\tau }_{3}(j\omega )}^{{\psi }_{3}}}$$
  where $R$ is the series resistor’s value,${R}_{i}$ is the resistor, ${\tau }_{i}$ is the relaxation time and ${\psi }_{i}$ is the distribution coefficient of the relaxation time in the ZARC-Cole elements, where $i$ has a value of 1 or 2. ${\tau }_{3}$ and ${\psi }_{3}$ are the values of the parameters in the CPE. CPE was included in the model to consider the polarization impedance of the plant electrode at low frequencies.

In Repo's research on hydroponic willows, the circuit between the electrodes consisted of a stem, the interface between the root and the bud in the culture medium, the culture medium and the contact layer on the electrode surface. The Impedance Spectrum of this entity is composed by circuit modeling of a resistor with two ZARC Cole elements and a constant phase element (CPE) in series [40]. The polarization impedance of plant electrode interface at low frequency is simulated by CPE. When the electrode polarization impedance is considered in the model, the biological system can be more accurately simulated [40].

Cao and Repo set up the equivalent circuit model of a single root, model consists of two R/C series circuits: one R/C circuit represents the branch above the solution, consisting of a resistor $Rsa$ and a capacitor $Csa$, the other R/C circuit represents the root part immersed in the solution and is composed of three parts [68].

The root-soil system is a challenging subject for EIS because it contains multiple polarized interfaces with conducting media in the middle. A detailed electrical model of such a system consists of several linear circuit elements (resistors and capacitors) [22]. In addition to the root system itself, the EIS characteristics of the system are also affected by several experimental factors such as soil type, SWC, electrode type, spacing and position [40].

In earlier studies, the moisture content of the root-soil system had a strong effect on capacitance [20, 22]. In measuring electrical impedance data for potted tomatoes, Ozier-Lafontaine and collaborators have established the equivalent circuit of the lumped model, which is based on a series circuit (soil-roots-stem-electrode) of four resistors R/capacitor C parallel circuits and a series resistor $R0$ describing the soil. The results show that the root growth is characterized by the change of capacitance and resistance. At this stage, however, it is not possible to attribute changes in EIS to changes that occur at the soil root interface or in the medium within the root system during root development. An independent discussion of the environment in which plants grow is needed [24].

#### Parameter fitting

After the appropriate equivalent circuit model is determined, the fitting of model parameters is the most important step. Complex nonlinear least squares (CNLS) data fitting can be used to describe good properties of material-electrode systems [121]. This technique was first applied to impedance spectroscopy analysis in 1977.

The electrical impedance data of the root system are obtained by EIS measurement, mainly including the real part, imaginary part, impedance Angle and other direct parameters. Through the CNLS method, we can also fit a series of derivative parameters such as the intracellular resistance and extracellular resistance, which are also very important data materials for us. [122, 123]. In addition, CNLS can fit very complex models, even if these models are very complex models with more than 10 unknown parameters [114].

Li Xingshu used the EIS method to detect kiwifruit pulp and used CNLS to calculate and fit the parameters $Ra$,$Rs, Zm$, and in the Hayden model. SPASS analysis software was used for difference significance analysis. Zhang Bo studied the effect of temperature change on apple tissue structure and applied CNLS to curve fitting of spectral data in the process of data processing [124]. Zhang used curves from CNLS for parameter estimation of equivalent circuit models [62, 63, 114]. After measuring EIS data of the hydroponic willow root system [40], model parameters were estimated using the CNLS curve fitting program LEVM 7, which was designed by Prof. J. R. MacDonald of North Carolina State University, USA. The program will get the minimized sum value:

5 $$S\left(Q\right)={\sum }_{j=1}^{N} {w}_{j}{\left[{X}_{j}-X{C}_{j}(Q)\right]}^{2}$$

where $N$ is the number of data points, ${w}_{j}$ is the weight related to the $j$ th point, ${X}_{j}$ is the value of the $j$th fitting data point and ${XC}_{j}(Q)$ is the value of the calculated fitting function involving the set of parameters $Q$. Parameters are very important in the initial selection, they must be as close to the real value as possible [47].

Based on published papers, CNLS is used in almost all studies on parameter fitting, and most researchers use J. R. Macdonald's LEVM series software for parameter fitting.

In Repo's study, model parameters are estimated to produce a total of nine parameters, namely series resistance $R$, distributed resistance ${R}_{1}$, relaxation time ${\tau }_{1}$, relaxation time distribution coefficient ${\psi }_{1}$, distributed resistance ${R}_{2}$, relaxation time ${\tau }_{2}$, distribution coefficient of relaxation time ${\psi }_{2}$, CPE relaxation time Constant ${\tau }_{3}$, CPE relaxation time distribution coefficient ${\psi }_{3}$ [39, 40]. Ozier-lafontaine established an equivalent model in tomato pot experiments, which had nine parameters whose values were fitted by LEVM 7 [24]. Zhang and collaborators found that the EIS of the European red pine stem was suitable for the double-DCE model, and there were 7 parameters to be measured. They were three resistances ($R$, ${R}_{1}$, and ${R}_{2}$), two relaxation times (${\tau }_{1}$ and ${\tau }_{2}$), and two distribution coefficients at different relaxation times (distribution coefficient, ${\psi }_{1}$, and ${\psi }_{2}$) [117]. CNLS curve fitting program LEVM8.09 was used to estimate the equivalent circuit parameters in the study of Cao, Y, and Repo, T. [68]. Cseresnyés measured the electrical impedance, phase Angle, and dissipation coefficient of the root system of pot maize with HP 4284A LCR-Bridge at a frequency range of 100 Hz ~ 10 kHz and a terminal voltage of 1 V [71]. Repo used the CLAFIC algorithm to calculate the EIS of Scotch pine seedlings treated with long-day and high temperature (LDHT) and short-day and low temperature (SDLT), and calculated the corresponding correlation coefficients. Then, in 2016, Repo and collaborators applied small-amplitude currents at 44 frequency points, ranging from 5 Hz to 100 kHz, to the root system during EIS measurements. At 5℃, − 5 ℃, − 12 ℃ and − 18 ℃, there were 830 real and imaginary parts of EIS in 2 cold acclimatizations and 3 mycorrhizal treatments roots [70, 74]. In the measurement of electrical impedance spectra, in addition to the real and imaginary data of electrical impedance, the impedance loss coefficient $\delta$ is introduced [69]. The impedance loss coefficient $\delta$ is calculated as $\delta =\left(\frac{{Z}_{Im}}{{Z}_{Re}}\right)$, and the frequency is selected as the best resolution between healthy and declining roots.

**Fig.7:**
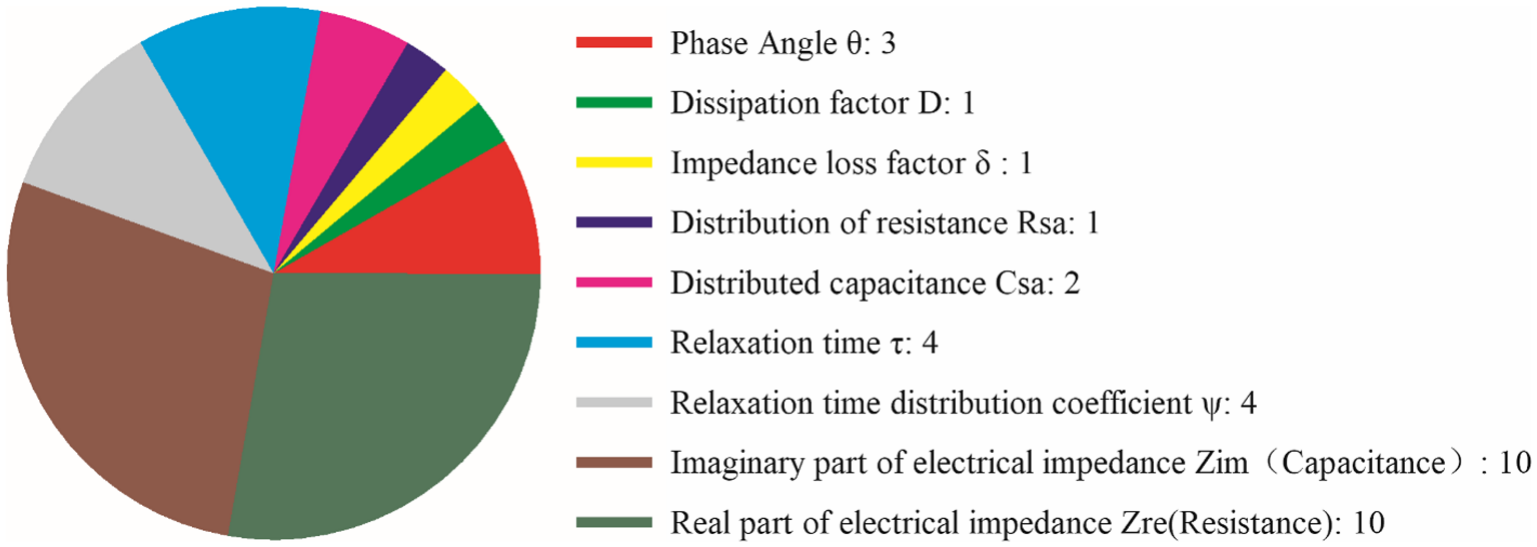
Statistics of EIS parameters and derived parameters

To sum up, all the references mentioned in this paper reported the corresponding research methods, as well as electrical impedance spectrum parameters testing, through summarizing, we can see the distribution parameters of the electrical impedance spectrum of literature mentioned, as shown in Fig. 7. The real part and imaginary part parameters of electrical impedance are most used, in addition, the more is phase Angle, loss factor, impedance loss coefficient, etc. In addition to the parameters mentioned above, we can also measure other characteristics of the electrical impedance research object, such as plant height, stem diameter, and other parameters. In the later stage, we can use artificial intelligence technology to combine the electrical impedance value with other parameters to jointly explore the influence of these composite parameters on plant growth. We can use compound parameters appropriately in future studies, or we can explore more new parameters that affect crop root characteristics, which may be more representative.

## The application of EIS and AI in roots

### Application field of EIS in roots

During the measurement of ex situ roots, EIS technique has been used to study the effect of freeze–thaw damage on electrical properties of potato tuber and carrot root tissues [61–63]. waterlogging stress of birch root [66], measurement of pM-ATpase activity and EIS in roots of *syringa oblata lindl*. Seedlings under saline-alkali stress [65], the cold resistance of the root system of different varieties of Rose [64], effects of cadmium stress on root growth, and EIS parameters of *cotinus coggygria* [67]. The measurement of ex situ roots requires destructive sampling of the root system, and the measurement process can only reflect the change of electrical impedance at the one-time point. Relevant literature shows that in situ measurements of the plant root system have been widely used in research [23, 50, 125–129].

In the past few years, the rapid development of sensor technology, artificial intelligence, and big data analysis technology has made the application of EIS more widely available. More and more multi-frequency response schemes have been used to measure the resistance and capacitance values of plant tissues. More comprehensive and novel root parameters can be obtained by the multi-frequency measurement EIS method. There is little literature on multi-parameter root studies based on the EIS technique, and the technique has not been used to quantify root growth, nor has the data analysis related to the equivalent circuit model of root system been carried out [128].

**Fig.8:**
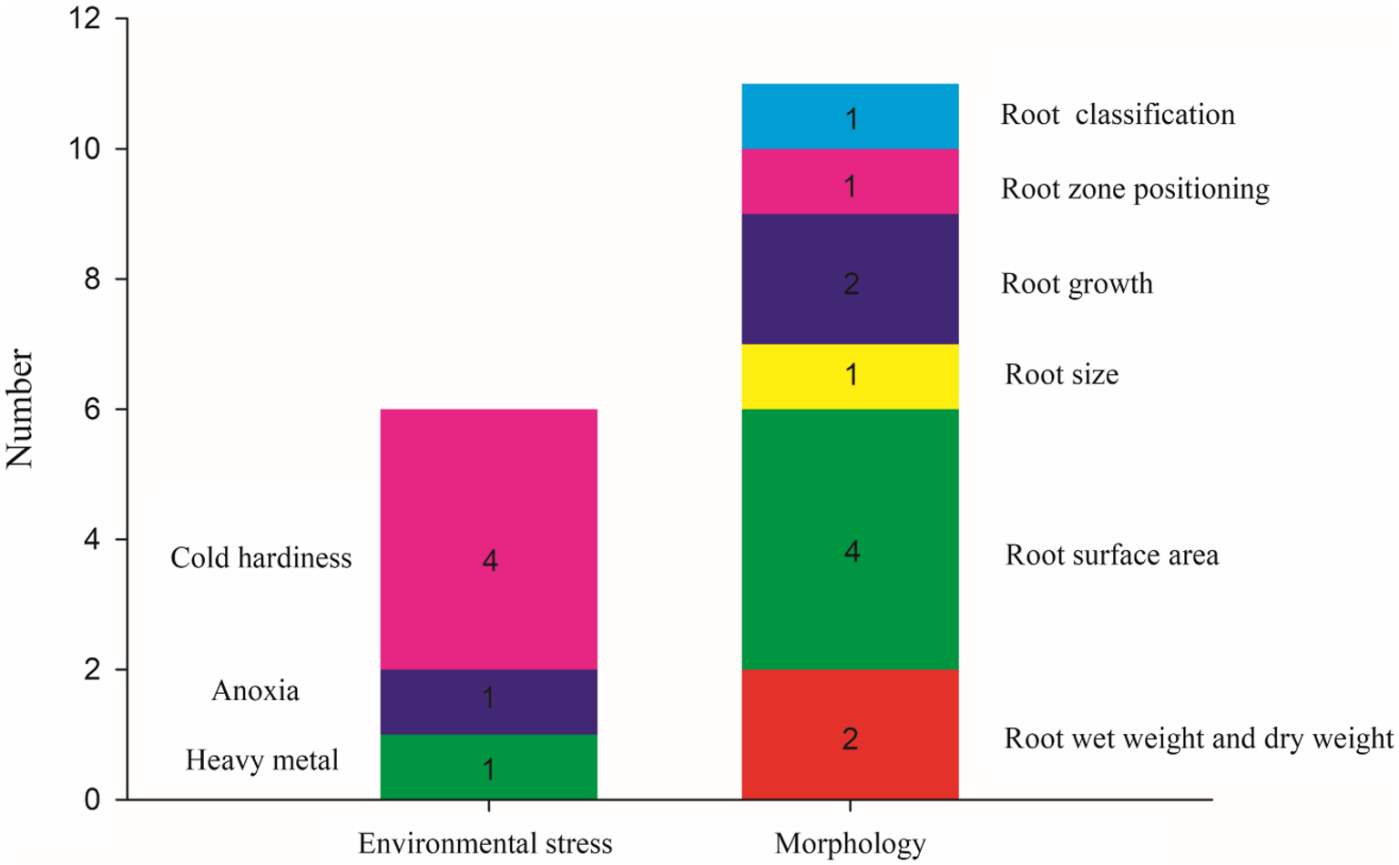
The number of papers in different root research areas using EIS

Many researchers have done a lot of work in the measurement of in situ roots. In this review, a total of 17 papers conducted EIS measurements on in situ roots systems, including 4 studies on plant cold resistance, 1 was used for anoxia stress and 1 was used for heavy metal stress. 11 studies were used for morphological study of plant roots, among which 2 were used for measuring the dry and fresh weight of roots, 4 were used for estimating root surface area, 1 was used for root size, 2 were used for root growth, 1 was used for root location, and 1 was used for root system classification. Figure 8 shows the number of papers in different root research areas using EIS.

#### Measurement of morphology parameters

As shown in Fig. 8, morphological is the focus of EIS technique in root research. In 1995, Dalton proposed a conceptual model to explain the root-soil system of plants, in which the root surface area was considered to be the surface area of a cylindrical condensers with the same average diameter as the root cell system. The capacitance of the root-soil interface was proportional to the accumulated charge on the surface of the active cell membrane. Therefore, the polarized membrane acted as a dielectric in the capacitor, with plant solution providing the first plate and soil solution providing the second plate [22]. Current was transferred from the soil to the roots through conductive water and ion absorption bands (and vice versa), so the electrical impedance values measured in the root-soil system were inversely correlated with active root surface area [24, 130]. Other researchers also found that measured root electrical parameters were directly related to root mass, root length, or root surface area [19, 128].

Repo measured the growth of willow roots by impedance spectroscopy, and research has shown that the EIS has the potential of monitoring the root growth and development. The method is not destructive to plants, and only minor tissue damage occur when the plant electrode is inserted into the stem; therefore, measuring root growth is quick and easy to perform. However, more research on the medium in which plants grow is needed in the future to find out the effects of soil type, soil moisture content, and electrode location on EIS parameters [39]. Ozier-lafontaine studied the effects of soil electrode orientation, the distance between soil electrode and plant root, the depth of insertion into soil and the position of plant electrode on EIS through potted tomato (Lycopersicon Esculentum Mill). A new method based on the EIS technique was proposed. It was found that there was a significant relationship between root dry weight and length increment through changes of electrical parameters. This study also highlights the importance of using impedance spectroscopy with a wide frequency range instead of a single frequency to measure root systems. Butler measured the absorbing root surface area of Norway spruce using the EIS method. The results showed a strong correlation between absorbent root surface area and diameter at breast height (DBH) [73]. Cao used EIS to measure the root of willow poplar in hydroponic culture and proved that the EIS method is an effective method for nondestructive evaluation of root surface area. Compared with the single-frequency measurement method, the EIS method provides an improved method for the study of root-related parameters. If the effect of stem contact with the growth substrate can be eliminated, the measurement of electrical impedance will be more meaningful. For further study of the EIS technique, it is necessary to consider the difference in the resistivity between roots and soil when soil is used as the growth medium [68]. Rajkai carried out a pot experiment on live roots using the capacitance method. Measured root capacitance can give a clear indication of root system quality and length development. Special attention should be paid to the selection of measurement frequency when measuring the root capacitance. To explain the characteristics of root capacitance and soil capacitance, a dual dielectric capacitance model is proposed. However, its effectiveness needs to be further verified [23, 28]. I Cseresnyes studied the variation rule of phase Angle in the application of impedance spectroscopy by executing pot experiments with maize, and proposed that the most effective characteristic current frequency should be selected according to the phase Angle spectrum when measuring electrical impedance. When measuring root dry mass and root surface area, the correlations between electrical impedance and root dry mass and root surface area were all frequency dependent, but in regression calculation, the coefficient of determination of electrical impedance and root surface area was slightly lower than that of electrical impedance and root dry mass. I Cseresnyes also found that measuring the phase Angle helps to improve the accuracy and reliability of the EIS results, so the variation of the phase Angle with frequency and time should be considered when using the EIS. However, the influence of soil properties and plant properties on the phase Angle spectrum should be considered in the future to ensure the validity of the measurement data [71]. A positive correlation between electrical signals and root surface area was observed in Peruzzo et al. [80], which may account for the physiological correlation between root regions that promote current flow and hair roots that contribute most to root surface area.

#### Environmental stress detection

Related literature shows that EIS has been successfully used to detect changes in plant tissue structure caused by environmental stress. Stress causes changes in membrane lipid and protein composition, affects membrane structure, permeability and ion balance, and changes the membrane surface charge density, and thus changes the surface potential [30, 131, 132]. EIS can detect the hypoxia effect of cadmium stress and waterlogging in early development of pea seedlings [44]. These results suggest that the concentration-dependent increase in the symplastic resistance and the energy inhibition effect of hypoxia may also follow the EIS [53]. Repo carried out EIS measurements and classification analysis of root electrical impedance spectra to study new methods for root mycorrhizal colonization and cold acclimation. Class-featuring information compression (CLAFIC) method was used in the classification process, and the results showed that the root system was different under cold acclimation and mycorrhizal treatment [74]. In addition, the CLAFIC method was used to classify and analyze the roots with different degrees of freezing injury, and a new method was established to distinguish the affected and unaffected roots [70]. Di evaluated the overwintering root status of scots pine seedlings by measuring the EIS of the roots and found that the roots had a lower tolerance to temperature. The root classification analysis was performed using the same classification method (CLAFIC) as Repo. However, the applicability of this method needs to be further tested under different tree species, provenances, and different seedling systems [69].

In addition, to study root diseases, the researchers have developed a bioEIS system that uses a frequency range (1 kHz ~ 1 MHz) as the excitation source frequency to compensate for potential errors in off-line analysis for early warning of plant root diseases [133].

In conclusion, the EIS method has deepened the application of root capacitance measurement analysis, but most studies are still based on hydroponic conditions for cultivation and measurement, only a few in non-hydroponic environment. Based on the current research status, the response of multi-frequency electrical impedance measurement technology in soil or substrate should be explored in the future to find key indicators that directly reflect the characteristics of plant roots or environmental stress. At the same time, EIS measurement equipment needs to be improved. The future development direction of measurement equipment is intelligence, miniaturization, portability and low cost. The measurement process can be more intensive, and the data monitored in real time can reflect the physiological changes of the plant in more detail. In the analysis of measurement results, the latest artificial intelligence classification technology and intelligent optimization technology can be introduced, and some more meaningful parameters and indicators can be obtained through data mining, so as to provide technical support for root evaluation.

### Combination of EIS and AI

After measuring the EIS data of plants, more derived parameters can be obtained through the relevant fitting software. Combined with other morphological indicators of plants themselves, the amount of EIS data should be very large. With the relevant parameters of EIS, the latest artificial intelligence technology can be used to carry out big data analysis, data mining, feature extraction, classification prediction and other work on this basis.

#### Data dimension reduction

Dimension reduction is to use some mapping method to map the data points in the original high-dimensional space to the low-dimensional space. By dimensionality reduction, we hope to reduce the error caused by redundant information and noise, make the data simpler and more efficient, and improve the accuracy of the application. In many applications, dimension-reduction algorithm has become a part of data preprocessing. The mainstream dimension-reduction algorithm currently in use include principal component analysis (PCA), linear discriminant analysis (LDA), locally linear embedding (LLE), laplacian eigenmaps (LE), and so on.

The electrical impedance data of the root system had been obtained by EIS measurement, mainly for electrical impedance real part, imaginary part, impedance Angle, and other direct parameters. Through the CNLS method, we can also fit a series of derivative parameters such as intracellular resistance, extracellular resistance, and so on, but we got under different frequency in a different time is still a vast amount of data. In order to extract some of the most obvious parameters affecting plant root characteristics, data processing, data cleaning and data dimension reduction analysis must be performed.

PCA and Sparse PCA (SPCA) are the most widely used methods in the application of plant root data dimension-reduction algorithm. PCA can identify the main features from the data, rotate the data axes to the most important directions on the data angle (with the largest variance), and then determine the number of principal components to be retained through the eigenvalue analysis, and eliminate other principal components, to achieve data dimension reduction [134]. In particular, the PCA algorithm is the most favored by researchers.

Repo processed EIS experimental data of root mycorrhizal fungi in Scottish pine. By analyzing and comparing SDLT and LDHT, it is found that the PCA responses of real and imaginary parts of impedance vary by 13% and 27% respectively. The dielectric properties of the leaves were measured at frequencies ranging from 100 kHz, 30 MHz to 100 kHz, and then the spectral data were simplified using PCA [74]. XIE performed PCA between impedance and quality indexes by using SIMCA-P 11.5. Forty-six different frequencies from 0.01 to 300 kHz were analyzed along the myofibers and across the myofibers [135]. Wang Ruolin and collaborators performed principal component analysis on 11 electrical parameters of apples measured at 13 frequencies in the aspect of water core disease detection and applied principal component analysis combined with different classification models to conduct discriminant analysis of good and bad apples [136].

PCA seeks to maximize the intrinsic information of the data after dimension reduction and measures the importance of the projection direction by measuring the variance of the data in the projection direction. However, such projection does not greatly distinguish the data but may make the data points mixed and cannot be distinguished. This is also the biggest problem with PCA, which leads to poor classification performance in many cases. SPCA obtains the modified principal element through sparse loading coefficient, and its theory is established on the basis that PCA can be rewritten into a regression-type optimal value problem. In apple mildew disease nondestructive testing, the researchers used SPCA screen out 27 impedance parameters with non-zero loading coefficient in 14 main effective components [137]. In the application of apple freshness nondestructive testing, Cai et.al. uses SPCA technology to select 39 sparse principal elements, which lays a foundation for the following classification algorithm [134].

SPCA is developed based on PCA. It is realized by extracting sparse principal components based on PCA. However, SPCA is a linear classification tool and cannot deal with nonlinear data. In addition to PCA and SPCA algorithms, LDA is also a commonly supervised Linear dimension-reduction algorithm. Unlike PCA, which maintains data information, LDA is designed to make the data points after dimensional reduction as easily distinguishable as possible. LDA is finally transformed into a problem of finding matrix eigenvectors, which is similar to PCA. PCA and LDA are the most important linear dimensionality reduction algorithms, and LDA can also be used for dimensionality reduction in future research.

In addition, Khaled used computational intelligence to analyze dielectric spectra data for oil palm disease detection, three feature selection algorithms were considered, including support vector machine feature selection (SVM-FS), genetic algorithm (GA) and random forest (RF) [76]. In the following research, Khaled also proposed the importance of using GA for feature selection [138].

#### Classification and prediction

In terms of computational intelligence technology, principal component analysis is usually used for dimension reduction, and classification algorithm is also a kind of intelligent algorithm often used, mainly for plant seedling selection, fruit quality classification, plant disease classification and so on. [138, 139]. Some of the most frequently used classification algorithms include linear discriminant analysis (LDA), quadratic discriminant analysis (QDA), K-nearest neighbor (KNN), support vector machine (SVM), artificial neural network (ANN), and Naive Bayes (NB) [76, 137, 140].

In apple mildew disease nondestructive testing, support vector machine (SVM) and artificial neural network (ANN) were used as classifiers to identify mildew core disease [137]. Ruolin Wang et al. applied principal component analysis combined with Fisher discriminant, Multilayer Perceptron (MLP) artificial neural network and radical Basis function (RBF) classification model to perform discriminant analysis on apple quality. Especially, the combination of loss factor and MLP or radial basis function is the best. Therefore, the classification effect is significantly improved [136]. By means of sparse principal component analysis-Linear classifier (SPCA-LDC) model, Cai obtained the appropriate sample selection proportion for apple fruit classification, and the accuracy of apple fruit freshness classification reached the highest [134]. Khaled et al. used support vector machines (SVM) and artificial neural network (ANN) classification models in oil palm disease detection [76]. In 2020, Markku Tiitta et al. conducted classification research on pine heartwood content and birch bark content, and three machine learning methods were adopted in the research process: K-nearest neighbor (KNN), decision tree (DT) and support vector machine (SVM). By comparing the three algorithms, it is found that the combination of EIS and K-nearest neighbor classifier has the highest accuracy [141]. In Huh 's study, EIS was combined with meat images, and Adaboost classification and gradient enhanced regression algorithm were used to predict meat freshness [142]. After measuring EIS data, it makes a prediction for freshness of chicken meat through classification algorithm [143]. In the study of thyroid nodule classification, multiple EIS signal features were extracted and analyzed from the data set, and a multi-featureless Bayesian network was established to classify and predict thyroid nodule [144]. In Feng L 's study, by measuring the electrical impedance spectra of fresh and naturally aged rice seeds, Fisher linear discriminant analysis method was used to establish a seed prediction model with the impedance, phase Angle and three-dimensional size of seeds at selected frequencies as independent variables and fresh and aged seeds as dependent variables [145].

In recent years, EIS has been widely used as a preliminary nondestructive testing method. With the popularization of artificial intelligence technology, after measuring EIS data of plants, there are more and more literatures on data classification and prediction using computational intelligence methods. It is indicated that the combination of EIS technology and computational intelligence technology is the general trend, and is an important means of exploring various indicators of plant growth process in the future.

## Conclusions and prospects

The literature shows that EIS has been widely used in the plant, crop, and food science in recent years. The EIS of plant tissue can provide information about plant cell structure, water content, properties, and integrity of plasma membrane, intracellular and extracellular parts of plant tissue [105]. It is difficult to measure the crop root system quickly and accurately. EIS technology makes it possible to measure roots in situ, and the non-invasive nature of this technology applies to all types of plant tissues.

### Technical details of the measurement

Through the analysis and research of literature, we found that the measurement technical details can determine the accuracy and rationality of the measurement data, which is mainly manifested in the following aspects:

- In the selection of the measuring electrode, in addition to the clamp electrode when water is the medium, the needle electrode is generally selected when the medium is the substrate or soil.
- In the measuring system composed of electrodes and plants, the electrodes are generally placed 2 cm above the junction of plant roots and stems, and the accuracy of the measuring data will be affected if the position is too high or too low. For the other end of the electrode, a rod is usually placed at one end of the inner container if it is a nutrient solution. For substrate or soil, a stainless steel plate electrode with peaks is usually placed at the bottom of the container or pot. In addition, non-polarized electrodes are recommended to minimize the impact of electrode polarization on the measurement. One of the most common electrode types is the Ag–AgCl electrode [24].
- In the measurement frequency, generally choose a certain range of measurement spectrum, both low frequency, and high frequency. The general low frequency can be the best at 50 Hz ~ 80 Hz. If the conditions do not allow, the maximum can be up to 1 kHz. Generally, the high frequency can be selected to about 50 kHz, and the higher excitation frequency has little effect on the results.
- For the equivalent circuit model, there may be multiple circuit models that can fit the impedance data well, so there is the problem of non-uniqueness, so the equivalent circuit model is sometimes not very accurate. As a result, alternative methods of interpretation are increasingly used, such as PCA, fitting of empirical spectral models, or Debye decomposition of spectra [41].
- The combination of EIS technology and AI is the development direction of EIS research, and the efficient and reasonable use of EIS data needs the help of AI technology. AI is an important technical means to establish a relationship between EIS measurement results and plant physiological characteristics, morphological characteristics and environmental stress.

### The limitations of EIS method

At present, there are few studies on in situ root EIS measurement of plants, and the accuracy is low. Therefore, the influencing factors need to be further studied. This is mainly because it is difficult to determine the electrical properties of the root growth medium, especially the complex soil components, and the conductive factors are greatly affected by the soil components and soil moisture. Therefore, it is difficult to fully explain the electrical properties of the soil-root-electrode continuum, and we need to further study the effect of soil conductivity on the overall electrical properties so that the method can be applied under different environmental conditions [24]. In addition, it must be known that measurement of plant EIS usually includes not only the spectrum of the plant tissue, but also the impedance of the tissue between the electrodes, because the electrodes are often pierced into the tissue, causing local damage to the cells [62, 63]. Another disadvantage is that measurements of in situ root EIS in plants are strongly influenced by soil conditions (water saturation, texture and chemical composition) and plant electrode placement [28, 29, 36], so the measurements are comparable only when the same plant species are planted and measured under the same conditions (Homogeneous planting medium, constant soil moisture, salinity and temperature, consistent electrode placement, etc.) [21]. In addition, Urban[106], Peruzzo[80] et al. found that most charge carriers leave the root system at the proximal end of the root and radially penetrate into the surrounding growth medium, and electrical impedance measurement cannot provide reliable data on the absorption area of distal fine roots. These results confirm doubts about Dalton's model [25, 106], so applying this technique to naturally growing plant roots inevitably leads to misleading conclusions.

### Future research perspectives

Electrochemical impedance spectroscopy (EIS), as a simple, fast, repeatable, and nondestructive technique, plays an important fundamental role in electrochemistry and biological sciences. Modern impedance analyzers have made it possible to steadily improve the study of applied impedance in plant science by extending the frequency range from millihertz to megahertz [146]. In situ nondestructive measurement of crop roots by EIS provides a new way for current and future researchers to study crop roots. But the technique still has many shortcomings in detecting crop roots. It is suggested that future research should be carried out from the following aspects.

At first, with the rapid development of embedded technology and artificial intelligence, we can customize a new type of EIS measurement system, which can be smaller and more sophisticated, easier to operate, less costly, and can also be placed in the measurement site. In a certain period, the continuous measurement of the changes of plant root electrical impedance can be used to analyze the relationship between the changes of electrical impedance and physiological indexes from a more microscopic point of view, which lays a good foundation for the more accurate study of root growth.

Secondly, it is necessary to further study the path of the current in the root system and the conductive mechanism of the contact layer between the root system and the medium, and combine the electrochemical knowledge to study the standardized root soil and water conditions required based on the EIS. In addition, other electrical technologies and intelligent methods can be combined to detect more accurate changes in root distribution and soil moisture content, providing corresponding technical support for future cultivation and breeding.

Thirdly, although the EIS technique has made significant progress in the process of in situ root measurement, the results of EIS measurement have been questioned due to the many disturbing factors in the growing medium, especially in soil. However, we can pay less attention to the detailed EIS measurements of plants as they grow, and pay more attention to the changes in the EIS measurements of plants over time. Big data technology and AI technology combined with EIS measurement data can be used to infer plant physiology, plant morphology, plant environmental stress or pests and diseases, which is more meaningful for our future EIS research.

EIS is a useful tool to evaluate physiological changes during plant development, especially after underground roots are subjected to certain stresses. For in situ root measurements, the successful application of the EIS technique requires special attention to the standardization of the composition of the growth medium, the scientificity of the measurement scheme, and the combined use of AI methods.

## Data Availability

Not applicable.

## Abbreviations

EIS: Electrical Impedance Spectroscopy
NDT: Nondestructive Testing
EC: Electrical capacitance
GPR: Ground Penetrating Radar
NMR: Nuclear Magnetic Resonance
ISMT: Impedance Spectroscopy Measuring Technology
AC: Alternating Current
ACG: Alternating Current Generator
LCR: Inductance, Capacitance, Resistance
CLAFIC: Class-Featuring Information Compression
DCE: Distributed Circuit Element
CNLS: Complex Nonlinear Least Squares
PCA: Principal Component Analysis
LDA: Linear Discriminant Analysis
LLE: Locally Linear Embedding
SPCA: Sparse PCA
SVM: Support Vector Machine
ANN: Artificial Neural Network
CPE: Constant Phase Element
DBH: Diameter at Breast Height
AI: Artificial Intelligence
LDHT: Long-day and high temperature
SDLT: Short-day and low temperature
SWC: Soil water content
QDA: Quadratic discriminant analysis
KNN: K-nearest neighbor
NB: Naive Bayes
MLP: Multilayer perceptron
RBF: Radical basis function
SPCA-LDC: Sparse principal component analysis-Linear classifier
DT: Decision tree
SIP: Spectral induced polarization
EIT: Electrical impedance tomography
